# Preoperative decompression for left-sided obstructive colorectal cancer: a comparative study between a stent and a decompression tube

**DOI:** 10.20452/wiitm.2025.17956

**Published:** 2025-07-04

**Authors:** Xin‑Chun Guo, Qiang Lu, Yu‑Fei Fu, Xin Lu

**Affiliations:** Department of Interventional Radiology, Jiangyin Hospital Affiliated with Nantong University, Jiangyin, China; Department of Radiology, Xuzhou Central Hospital, Xuzhou, China

**Keywords:** colorectal cancer, decompression tube, obstructive, oncology, stent

## Abstract

**INTRODUCTION:**

Stents and decompression tubes (DTs) are both commonly utilized for preoperative decompression in patients with left-sided obstructive colorectal cancer (OCRC). However, the comparative effectiveness and oncological safety of these 2 approaches remain uncertain.

**AIM:**

The aim of this study was to compare clinical performance and long-term oncological outcomes of preoperative stent and DT insertion in patients with left-sided OCRC.

**MATERIALS AND METHODS:**

The study included 87 consecutive patients, diagnosed with left-sided OCRC between January 2022 and December 2024. All patients underwent preoperative decompression using either a stent or a DT. Clinical decompression efficacy, surgical outcomes, and oncological parameters were compared between the groups.

**RESULTS:**

Among the 87 patients enrolled, 45 received a stent and 42, a DT. Technical success rates were similar between the groups (stent, 95.6% vs DT, 97.6%; P >0.99), as were clinical success rates (stent, 88.9% vs DT, 90.5%; P >0.99). Perforation occurred in 3 patients (6.7%) in the stent group and 1 participant (2.4%) in the DT group (P = 0.62). Primary anastomosis was achieved in 84.4% of the stent patients and 81% of the DT patients (P = 0.67). Median disease-free survival was 32 (21–43) months in the stent group and 34 (25–43) months in the DT group (P = 0.35), while median overall survival was 38 (29–47) and 39 (34–44) months, respectively (P = 0.53), with no apparent differences between the groups.

**CONCLUSIONS:**

Both stent and DT insertion are safe and effective strategies for preoperative left-sided OCRC management. No significant differences in either short-term clinical outcomes or long-term oncological results were observed between the 2 methods.

## INTRODUCTION

Bowel obstruction is a common complication in colorectal cancer, affecting approximately 10%–20% of patients,^1^^,^^2^^,^^3^ with the majority (around 70%) of acute obstructions attributed to tumors located in the left colon.^4^ Traditionally, emergency surgery has been the standard strategy for managing obstructive colorectal cancer (OCRC).^5^ However, such procedures are linked to high morbidity and mortality, as well as a significant risk of temporary or permanent stoma formation.^6^ To mitigate these risks, preoperative decompression is increasingly employed to stabilize patients, improve the likelihood of primary anastomosis, and reduce perioperative complications and mortality.^6^

Currently, self-expanding metal stents and colonic decompression tubes (DTs) are the most widely used modalities for preoperative decompression in left-sided OCRC. Both techniques effectively relieve colonic obstruction, facilitating elective rather than emergency surgical intervention.^7^^,^^8^^,^^9^ Despite their widespread use, direct comparisons between these 2 methods, particularly with respect to long-term oncological outcomes, remain limited.^10^^,^^11^^,^^12^^,^^13^

## AIM

This study aimed to compare clinical effectiveness and oncological outcomes of preoperative decompression using stents vs DTs in patients with left-sided OCRC.

**TABLE 1:** The ColoRectal Obstruction Scoring System

Level of oral intake	Score
Requiring continuous decompression	0
No oral intake	1
Liquid or enteral nutrient intake	2
Soft solids, low-residue, and full diet with symptoms of stricture	3
Soft solids, low-residue, and full diet without symptoms of stricture	4

### MATERIALS AND METHODS

#### Study design

This was a retrospective, observational study conducted at 2 medical centers. Institutional review board approval was obtained from the Ethics Committees of Jiangyin Hospital (20250205) affiliated with Nantong University and Xuzhou Central Hospital. Due to the retrospective nature of the study, informed consent requirements were waived. Patients who were treated between January 2022 and December 2024 for left-sided OCRC and underwent preoperative decompression via a stent or a DT were considered eligible. Inclusion criteria were: histologically confirmed left-sided OCRC and ColoRectal Obstruction Scoring System score between 0 and 2 (TABLE 1). Exclusion criteria comprised clinical T4b stage, inability to undergo sphincter-preserving surgery, American Society of Anesthesiologists score higher than or equal to 4, and a presence of distant metastasis.

### Diagnosis

Diagnosis of left-sided OCRC was established based on clinical presentation, contrast-enhanced computed tomography (CT), magnetic resonance imaging (MRI), and endoscopy. Cases were defined by a tumor located between the splenic flexure and the rectum.^1^ All patients also underwent chest CT, brain MRI, and bone scintigraphy to assess for distant metastases.

### Decompression procedure

Decompression was performed under fluoroscopic guidance using a transanal approach. A 5-F VER catheter (Cordis, Hialeah, Florida, United States) and a 0.035-inch hydrophilic guidewire (Terumo, Tokyo, Japan) were employed to identify the obstruction site. Once the site was located, the guidewire was withdrawn and a contrast medium was injected to delineate the obstruction. After that, a 0.035-inch stiff guidewire (Cook Medical, Bloomington, Indiana, United States) was inserted through the catheter which was then removed.

In the stent group, a self-expanding metal stent (Micro-Tech, Nanjing, China) was deployed over a stiff guidewire. The stent length exceeded that of the obstruction by at least 30 mm (15 mm beyond each margin). The diameter of the stent was 30 mm.

In the DT group, a 22-F colonic decompression tube (Create Medic Co., Dalian, China) was placed and secured with a 30-ml water-filled balloon at the tip. The patients received colonic irrigation 4 times daily for 5 to 7 days following placement.

### Surgical resection

Elective surgery was scheduled approximately 1 week after successful decompression, contingent upon symptom relief and radiographic improvement. Surgical resection, with or without protective stoma creation, was performed at the discretion of the operating surgeon, based on intraoperative findings. The patients with unsuccessful decompression underwent emergency surgery, typically involving resection and stoma formation.

### Assessment

Technical success of the stent placement was defined as a complete coverage of the obstruction, while for DT insertion, it was defined as a correct positioning of the tip proximal to the obstructed segment. Clinical success was defined as the resolution of obstruction symptoms and radiologic improvement within 48 hours after the procedure.

The primary end point was successful clinical decompression. Secondary end points included technical success, procedure-related complications, primary anastomosis rates, postoperative complications, and long-term outcomes comprising overall and disease-free survival (OS and DFS).

The patients underwent follow-up at 3, 6, and 12 months postoperatively, and after that, at 6-month intervals. Follow-up assessments included physical examination, imaging, and carcinoembryonic antigen testing. Data collection ended in March 2025 or upon patient’s death.

### Statistical analyses

Continuous variables were reported as mean (SD) when normally distributed, and, if skewed, as median (interquartile range [IQR]). Intergroup comparisons were performed using the *t* tests or the Mann–Whitney tests, as appropriate. Categorical variables were analyzed via the χ^2^ test or the Fisher exact test. Survival outcomes were evaluated using the Kaplan–Meier curves and log-rank tests. A *P* value below 0.05 was deemed significant. Analyses were conducted with IBM SPSS Statistics package, version 16 (IBM Corp., Armonk, New York, United States).

## RESULTS

### Patients

The study included 87 patients diagnosed with left-sided OCRC who underwent preoperative decompression with the use of either a stent (n = 45) or a DT (n = 42). Baseline characteristics of the 2 cohorts were comparable (TABLE 2).

### Decompression outcomes

Technical success was achieved in 95.6% (43/45) of the patients in the stent group and 97.6% (41/42) of the DT group participants, with no significant differences (*P* >0.99; TABLE 3). In both groups, failure was attributed to an inability to navigate the obstructed segment with a catheter and a guidewire. Clinical success was similarly high: 88.9% (40/45) for the stent placement and 90.5% (38/42) for the DT insertion (*P* >0.99). In the stent group, clinical failure in 5 patients was due to technical failure (n = 2) and bowel perforation (n = 3). Among the 4 clinical failures in the DT group, the causes included technical failure (n = 1), perforation (n = 1), and insufficient fecal drainage (n = 2). Postprocedural perforation occurred in 6.7% (3/45) of the stent group and 2.4% (1/42) of the DT group (*P* = 0.62).

**TABLE 2:** Baseline patient data

Parameter		Stent group (n = 45)	Decompression tube group (n = 42)	P value
Age, y, mean (SD)		63.6 (12.5)	64.6 (9.7)	0.67
Sex, n (%)	Men	25 (55.6)	24 (57.1)	0.88
	Women	20 (44.4)	18 (42.9)	
Tumor location, n (%)	Colon	28 (62.2)	26 (61.9)	0.98
	Rectum	17 (37.8)	16 (38.1)	
CEA, μg/l, median (IQR)		2.2 (1.3–6.2)	1.6 (1–3.2)	0.22
ASA score	1	10 (22.2)	11 (26.2)	0.84
	2	22 (48.9)	18 (42.9)	
	3	13 (28.9)	13 (30.9)	
T stage, n (%)	2	7 (15.6)	6 (14.3)	0.66
	3	25 (55.6)	20 (47.6)	
	4	13 (28.8)	16 (38.1)	
N stage, n (%)	Positive	18 (40)	19 (45.2)	0.48
	Negative	27 (60)	23 (54.8)	
Differentiation, n (%)	Good	8 (17.8)	6 (14.3)	0.83
	Moderate	25 (55.6)	26 (61.9)	
	Poor	12 (26.6)	10 (23.8)	

Abbreviations: ASA, American Society of Anesthesiologists; CEA, carcinoembryonic

antigen; IQR, interquartile range

**TABLE 3:** Decompression and surgical outcomes

Parameter		Stent group (n = 45)	Decompression tube group (n = 42)	P value
Technical success of decompression		43 (95.6)	41 (97.6)	>0.99
Clinical success of decompression		40 (88.9)	38 (90.5)	>0.99
Decompression-related perforation		3 (7.7)	1 (2.4)	0.62
Type of surgery	Emergency	5 (11.1)	4 (9.5)	>0.99
	Elective	40 (88.9)	38 (90.5)	
Primary anastomosis		38 (84.4)	34 (81)	0.67
Postoperative complications	Abdominal abscess	2 (4.4)	1 (2.4)	>0.99
	Leakage	2 (4.4)	2 (4.8)	>0.99
	Ileus	3 (6.7)	2 (4.8)	>0.99
	Wound infection	0 (0)	1 (2.4)	0.48

Data are presented as number (percentage).

### Surgical approach

In the cases where decompression was successful, elective surgery was performed, while the patients with clinical failure required emergency intervention. R0 resection was successfully carried out in all cases. There were no significant discrepancies in tumor staging (T and N) or histological differentiation between the 2 groups (TABLE 2). Primary anastomosis was achieved in 84.4% (38/45) of the patients in the stent group and 81% (34/42) of the DT group participants (*P* = 0.67; TABLE 3).

Postoperative complications were similar in both groups (TABLE 3). In the stent group, they included abdominal abscess (n = 2), anastomotic leakage (n = 2), and ileus (n = 3), while in the DT group, abdominal abscess (n = 1), anastomotic leakage (n = 2), ileus (n = 2), and wound infection (n = 1).

### Follow-up

**FIGURE 1:**
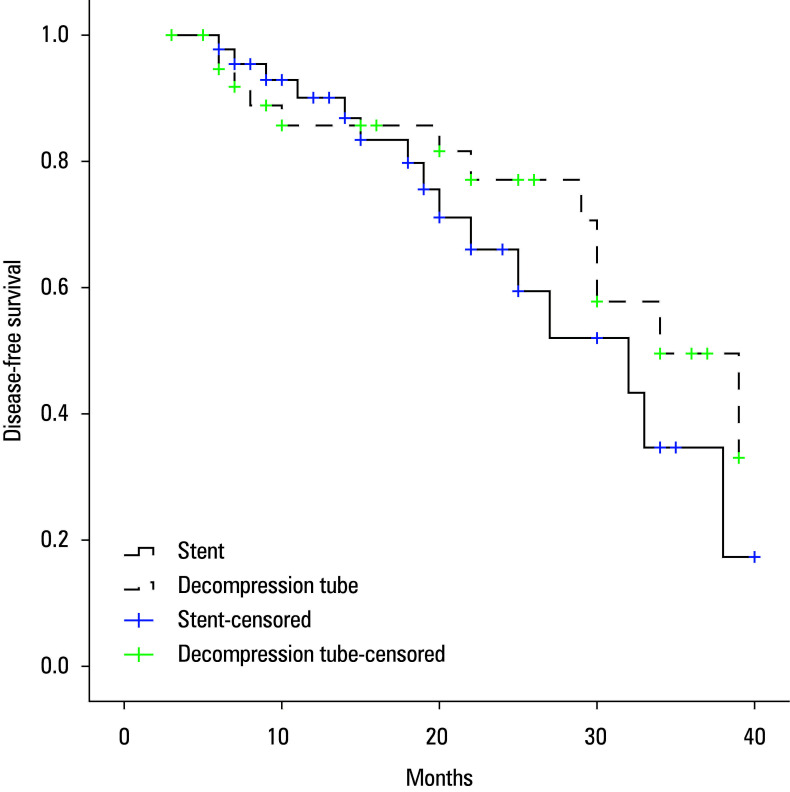
Disease-free survival rates in both groups (log‑rank test; P = 0.35)

**FIGURE 2:**
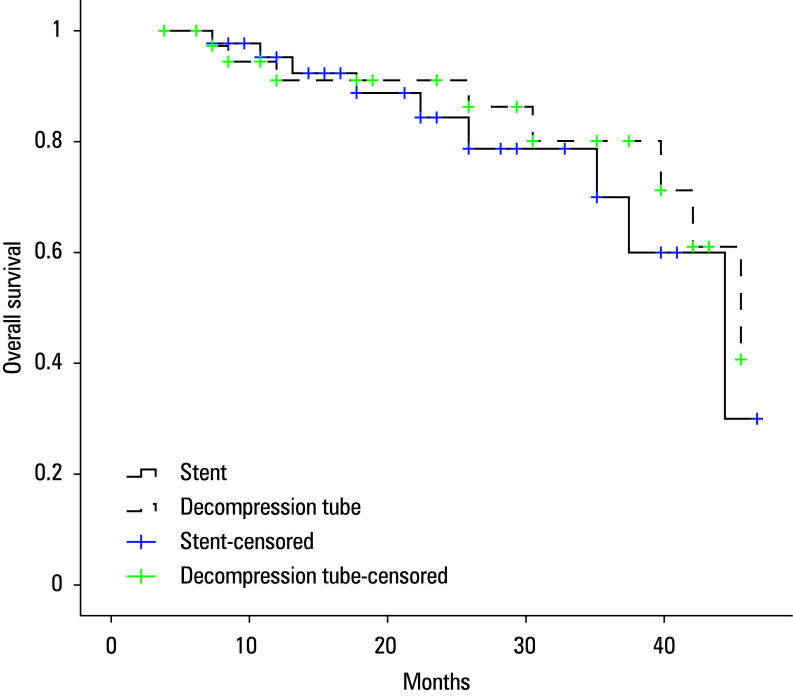
Overall survival rates in both groups (log‑rank test; P = 0.53)

Median follow-up was 18 (3–40) months. All individuals with stage III disease received adjuvant chemotherapy. Stoma reversal was performed in 4 out of 7 patients (57.1%) in the stent group and 5 out of 8 patients (62.5%) in the DT group (*P* >0.99). Reasons for nonreversal included tumor progression (n = 2) and a poor general condition (n = 6). Tumor recurrence or distant metastasis was observed in 7 patients (15.6%) in the stent group and 6 participants (14.3%) in the DT group. Median DFS was 32 (21–43) months for the stent group and 34 (29–47) months for the DT group (*P* = 0.35). One-year and 3-year DFS rates were 90.1% and 34.7% for the stent group, compared with 85.7% and 49.5% for the DT group, respectively (FIGURE 1). During follow-up, 10 patients in the stent group and 8 patients in the DT group died. Median OS was 38 (29–47) months and 39 (34–44) months for the stent and DT groups, respectively (*P* = 0.53). One-year and 3-year OS rates were 92.3% and 60% in the stent group, and 91.1% and 61% in the DT group, respectively (FIGURE 2).

### Subgroup analysis

There were 28 colon cancer patients in the stent group and 26 in the DT group. Technical success rates of decompression were 96.4% (27/28) and 96.2% (25/26), respectively (*P* >0.99*)*. Clinical success rates were 89.3% (25/28) and 88.5% (23/26), respectively (*P* >0.99). Primary anastomosis rates were 85.7% (24/28) and 80.8% (21/26), respectively (*P* = 0.72). Median DFS was 27 (19–34) months for the stent group and 39 (29–46) months for the DT group (*P* = 0.39). One-year and 3-year DFS rates were 96.3% and 16.6% for the stent group, and 85.3% and 59.7% for the DT group, respectively. Median OS was 38 (33–44) months and 39 (31–46) months for the stent and DT groups, respectively (*P* = 0.78). One-year and 3-year OS rates were 96.3% and 53.5% in the stent group, and 89.8% and 60% in the DT group, respectively.

There were 17 rectal cancer patients in the stent group and 16 in the DT group. Technical success rates of decompression were 94.1% (16/17) and 100% (16/16), respectively (*P* >0.99). Clinical success rates were 88.2% (15/17) and 93.8% (15/16), respectively (*P *>0.99). Primary anastomosis rates were 82.4% (14/17) and 81.3% (13/16), respectively (*P* >0.99). Median DFS was 38 (30–42) months for the stent group and 30 (24–37) months for the DT group (*P* = 0.6). One-year and 3-year DFS rates were 81.9% and 55.3% for the stent group, and 85.7% and 34.3% for the DT group, respectively. Median OS was 38 (32–45) months and 39 (32–46) months for the stent and DT groups, respectively (*P* = 0.26). One-year and 3-year OS rates were 87.4% and 68.8% in the stent group, and 92.9% and 61.9% in the DT group, respectively.

## DISCUSSION

Emergency surgery for OCRC is associated with substantial risk, with reported mortality rates of 15%–34% and morbidity ranging from 32% to 64%.^1^ Preoperative decompression strategies, such as stent placement or DT insertion, offer a means of converting emergency cases into planned elective procedures, potentially reducing perioperative risk.^14^^,^^15^^,^^16^

This study directly compared clinical performance and long-term outcomes of stents and DTs as preoperative decompression methods. Technical success rates were similarly high in both groups (95.6% vs 97.6% in the stent vs DT group, respectively; *P* >0.99), likely reflecting procedural factors, such as the use of fluoroscopic guidance. There are several factors that may have been related to the high rates of technical success in this study: 1) the stents and DTs were placed under fluoroscopic guidance, 2) fluoroscopy helped visualize both the obstruction site and its length, using contrast media, and 3) a guide-wire was used for the placement of stents and DTs.

Clinical success rates were also high and comparable between the groups (88.9% vs 90.5% in the stent vs DT group, respectively; *P* >0.99), suggesting that both methods effectively relieve colonic obstruction. These results are in line with previous studies^6^**^,^**^13^**^,^**^17^ that reported clinical success rates for stent insertion in the range of 78.6%–85.2%. Despite a DT’s smaller caliber in comparison with a stent, its ability to facilitate bowel irrigation enhances stool softening and dilution, thereby improving drainage efficiency.

Perforation rates were similarly low in both groups (6.7% vs 2.4%; *P* = 0.62), highlighting the procedural safety of both techniques. Nevertheless, when perforation occurs, immediate emergency surgery is required.**^1^**^13^

One of the primary goals of preoperative decompression in OCRC is to increase the likelihood of primary anastomosis while minimizing operative risk. A meta-analysis previously demonstrated^18^ that preoperative stenting significantly improved the primary anastomosis rate in comparison with emergency surgery (67.2% vs 55.1%; *P* = 0.007). In this study, both decompression methods yielded high and comparable anastomosis rates (84.4% vs 81% in the stent vs DT group, respectively; *P* = 0.67), with similarly low postoperative complication rates. These favorable outcomes reflect the effective decompression achieved in both groups.

With respect to oncological outcomes, both DFS and OS were similar in both groups. This finding suggests that the choice of decompression method does not adversely affect long-term cancer prognosis in left-sided OCRC. Several previous studies^2^**^,^**^7^ have also reported comparable survival outcomes of these 2 techniques. Although Sato et al^8^ observed considerably better 3-year recurrence-free survival in the stent group (74.9% vs 40.9%; *P* = 0.003), their study was limited by an unbalanced sample (stent, n = 60 vs DT, n = 18), introducing potential bias and limiting generalizability. By contrast, our study enrolled nearly equal numbers of patients in both groups, strengthening the reliability of the findings.

Despite its strengths, this study has several limitations. First, its retrospective design renders it susceptible to selection bias. Second, the sample size was modest, which may have limited its statistical power. Third, the median follow-up duration of 18 months was relatively short, restricting our ability to evaluate long-term oncological outcomes.

## CONCLUSIONS

In summary, both self-expanding metal stents and DTs appear to be safe and effective options for preoperative bowel decompression in patients with left-sided OCRC. Importantly, the choice between these 2 techniques did not impact long-term oncological outcomes, supporting their interchangeability based on clinical context and expertise.

## JOURNAL INFORMATION

Videosurgery and Other Miniinvasive Techniques is an official journal of the Videosurgery Foundation.
